# Analysis of Antibiotic Use Patterns and Trends Based on Procurement Data of Healthcare Institutions in Shaanxi Province, Western China, 2015–2018

**DOI:** 10.3390/ijerph17207536

**Published:** 2020-10-16

**Authors:** Sen Xu, Shengfang Yuan, John Alimamy Kabba, Chen Chen, Wenchen Liu, Jie Chang, Yu Fang

**Affiliations:** 1 Department of Pharmacy Administration and Clinical Pharmacy, School of Pharmacy, Xi’an Jiaotong University, Xi’an 710000, China; marsxs@stu.xjtu.edu.cn (S.X.); f19980511@stu.xjtu.edu.cn (S.Y.); fudimamy@hotmail.com (J.A.K.); chenchen00@stu.xjtu.edu.cn (C.C.); liuwenchen@stu.xjtu.edu.cn (W.L.); 2 Center for Drug Safety and Policy Research, Xi’an Jiaotong University, Xi’an 710000, China; 3 Shaanxi Center for Health Reform and Development Research, Xi’an Jiaotong University, Xi’an 710000, China; 4 Research Institute for Drug Safety and Monitoring, Institute of Pharmaceutical Science and Technology, China’s Western Technological Innovation Harbor, Xi’an 710000, China

**Keywords:** trends and patterns, antibiotic utilization, healthcare institutions, west China

## Abstract

Overuse of antibiotics has caused a series of global problems, especially in the underdeveloped western regions where healthcare systems are fragile. We used antibiotic procurement data of all healthcare institutions to analyze the total amount, patterns and trends of antibiotic use in Shaanxi Province, western China between 2015 and 2018. Antibiotic utilization was quantified using the standard Anatomical Therapeutic Chemical (ATC)/Defined daily dose (DDD) methodology. The World Health Organization’s “Access, Watch and Reserve” (AWaRe) classification and European Surveillance of Antimicrobial Consumption (ESAC) drug-specific quality indicators were also adopted to evaluate the appropriateness and quality of antibiotic utilization. Overall, antibiotic consumption decreased from 11.20 DID in 2015 to 10.13 DID (DDDs per 1000 inhabitants per day) in 2016, then increased to 12.99 DID in 2018. The top three antibiotic categories consumed in 2018 were J01C (penicillins) 33.58%, J01D (cephalosporins) 29.76%, and J01F (macrolides) 19.14%. Parenteral antibiotics accounted for 27.41% of the total consumption. The largest proportion of antibiotic use was observed in primary healthcare institutions in rural areas, which accounts for 51.67% of total use. Consumption of the Access group, the Watch group, the Reserve group of antibiotics was 40.31%, 42.28% and 0.11%, respectively. Concurrently, the consumption of J01D and the percentage of J01 (DD + DE) (third and fourth generation cephalosporins) were at a poor level according to the evaluation of ESAC quality indicators. The total antibiotic consumption in Shaanxi Province had been on an upward trend, and the patterns of antibiotic use were not justified enough to conclude that it was rational. This is partly because there was high preference for the third and fourth generation cephalosporins and for the Watch group antibiotics.

## 1. Introduction

The irrational use of antibiotics is perhaps the single most important contributor to the global crisis of antimicrobial resistance (AMR) [1,2]. Overuse of antibiotics has caused a series of global problems, especially in middle-income and low-income countries [3,4]. A team of researchers headed by Jim O’Neill, a British scholar, predicts that if the current problem of antimicrobial resistance is not alleviated, by 2050, the estimated annual deaths caused by antimicrobial resistance will reach 10 million people, and the global GDP will shrink by 2.5%–3% [5].

At present, governments and societies of all nations have realized the harm caused by the unreasonable use of antibiotics, warranting the World Health Organization (WHO) in 2011 to propose to defend resistance: “no action today, no cure tomorrow [6].” The World Health Assembly in 2015 also reviewed and approved the global action plan to control AMR [7]; On the same issue, the G20 summits held in 2016 and 2017 included AMR as a major topic and it was written into the communiqué [8,9]; and at the 71st United Nations General Assembly, the issue of AMR was discussed by countries around the world, becoming the fourth health issue ever discussed by the UN General Assembly [10]. The growing threat of AMR to global public health requires action by all government agencies and societies [11].

China is a big producer and consumer of antibiotics [12]. An estimated 248,000 tons of antibiotics were produced in China in 2013 alone [13]. The Chinese government has made tremendous efforts in the fight against AMR. Among many interventions, it has implemented the Essential Medicines Policy to increase drug availability, the zero mark-up policy to reduce drug costs, and the special campaign of antibacterial drugs to supervise and inspect the use of antibacterial drugs nationwide [14,15,16]. In 2016, *China’s National Action Plan to Contain Antimicrobial Resistance (2016–2020)* was formulated and released, becoming one of the first countries to issue and implement an action plan [17]. The plan proposed to standardize and improve the clinical applications and management of antibiotics, establish effective antibacterial resistance monitoring networks, strengthen the training of relevant professional medical personnel, encourage the development of information dissemination, and expand publicity and education.

In line with the national agenda on the fight against AMR, the Health Commission of Shaanxi Province has issued the catalogue of hierarchical management of the clinical application of antimicrobial drugs [18]. This policy document emphasized that healthcare institutions’ application for antibiotics beyond the catalog will be subjected to approval only on sound and sufficient evidence-based medical evidence. They also carried out strict management in accordance with the principle of classified management as stated in the catalogue, so as to ensure the safety, effectiveness, and cost-effectiveness of antibiotics.

China’s policy has achieved reasonably positive results. According to the “Status Report on Antimicrobial Administration and Antimicrobial Resistance in China (2019) [19],” the usage rate of antimicrobials in hospitalized patients was 36.4% in 2018, decreased by 23% percentage points compared with that of 2011 (59.4%). Similarly, at the outpatient departments, the prescription rate was down by 8.3% compared with that of 2011 (17.2%). Moreover, the isolation rate of common clinical drug-resistant bacteria in China is on a downward trend or remains stable [19]. The current situation of antimicrobial use and bacterial resistance in China is the result of a holistic sustained top–bottom approach towards combating AMR.

Nonetheless, issues such as unbalanced development among different regions and healthcare institutions that are believed to exacerbate AMR, especially in the underdeveloped western regions where healthcare systems are relatively fragile, still prevail. As a pilot area for the WHO’s Western Area Health Initiative, the results of antibiotic use in Shaanxi Province will serve as models for other western regions and developing countries in the region.

Therefore, we used the Shaanxi Provincial Drugs and Medical Equipment Procurement Platform database to analyze the total amount, patterns and trends of antibiotic use in healthcare institutions in Shaanxi Province, western China. Furthermore, we evaluated the quality of antibiotic use, and compared the situation of Shaanxi Province with other regions. We believe recommendations from this study will be essential for improving the rational use of antibiotics.

## 2. Method

### 2.1. Study Setting

Shaanxi Province is located in western China, with a resident population of 38.64 million (2018) and a geographic area of 205,600 square kilometers. The urban and rural populations are 22.4638 and 16.1182 million, accounting for 58.13% and 41.87% of the total population, respectively [20]. In 2018, Shaanxi Province’s annual GDP was 2443.832 billion yuan, ranking 15th among 31 provinces across the country [21]. In 2017, the total health expenditure in Shaanxi Province was 153.805 billion yuan, accounting for 7.02% of its GDP, higher than the national average of 6.36% [22].

### 2.2. Data Source

Shaanxi Provincial Drugs and Medical Equipment Procurement Platform, affiliated with Shaanxi Public Health Administration Department, was responsible for centralised transactions in pharmaceutical and medical products procurement in healthcare institutions in Shaanxi Province. We extracted all antibiotic procurement data from 2015 to 2018 and analyzed the total amount, patterns and trends of antibiotic use in Shaanxi Province. The data includes all antibiotic procurement records from more than 350 hospitals and 1400 primary healthcare centers (PHCs) (Table 1).

The data captured by Shaanxi Provincial Drugs and Medical Equipment Procurement Platform includes the name of the healthcare institution, order time, chemical substance name, generic name, dosage form, strength, quantity purchased, expense, delivery time, etc. The names of healthcare institutions and manufacturers were not shown to ensure confidentiality.

### 2.3. Data Analysis

The procured medicines were categorized using the Anatomical Therapeutic Chemical (ATC) classification codes. Records of “J01” (antibacterials for systemic use) procurement were extracted and then included in the analysis. A total of 129 unique chemical substance names were identified in single or combination antibiotics. These antibiotics were aggregated into 29 ATC-4 classes and then into 10 ATC-3 groups. Antibiotic consumption was measured using the defined daily dose (DDD) developed by the World Health Organization (WHO) [23]. DDD is defined as the average daily dose maintained by adults with primary indications. This study used standardized drug unit DDDs, which is the concept of total antibiotic use. DDDs is the product of the sales package × number of drugs × strength of each drug/DDD. DID is an indicator of the intensity of antibiotic use in the population, which is DDDs per 1000 inhabitants per day. The inhabitants used here are the annual average resident population. The population information is obtained through the Statistical Bulletin of Economic and Social Development of Shaanxi Province.

The “Access, Watch and Reserve” (AWaRe) is a recent antibiotic classification in the Essential Medicines Model List published by WHO [24]. It is used to classify and calculate the drug usage patterns of different antibiotics groups. Antibiotics are classified into the Access, Watch, and Reserve group according to the levels that should be used with caution. We therefore adopted the AWaRe classification in analyzing antibiotic use in different groups from different healthcare institutions, and make a comparative analysis with other countries.

The 12 quality indicators measuring antibiotic use by the scientific advisory board of the European Surveillance of Antimicrobial Consumption (ESAC) project were adopted to assess the potential irrational use of antibiotics [25]. These 12 indicators measure the quality of antibiotic use from consumption, patterns and seasonal variation. The ESAC published the performance of 31 European countries on the 12 quality indicators from 2015 to 2018 to be used as benchmark [26]. We compared the antibiotic consumption in healthcare institutions in Shaanxi Province with 31 European countries during the same period.

Owing to the lack of the information system infrastructure, the data failed to cover all healthcare institutions, so we assumed that there is no systematic difference between the information contained and the information not included. When calculating the DID value, we adopted the following formula to calculate the adjusted consumption used in healthcare institutions to prevent underestimation of the use of antibiotics. In brief, we compared the number of healthcare institutions at all levels throughout the year in the statistical yearbook and used the proportion of each institution in Shaanxi to modify the data hierarchically. We used the following formula to calculate the DDDs used by a certain type of healthcare institution, and then added the DDDs used by six types of healthcare institutions, including tertiary hospitals, secondary hospitals, primary hospitals, ungraded hospitals, urban PHCs, and rural PHCs.

$$Yi=\sum_{i=1}^{6}Mi/\frac{ni}{Ni}$$

*Yi*: The adjusted DDDs in a given year in a certain type of healthcare institution ***i***;
*Mi*: DDDs used in a given year in a certain type of healthcare institution ***i***;
*ni*: Number of a certain type of healthcare institution ***i*** in the procurement platform;
*Ni*: The total number of a certain type of healthcare institution ***i*** in the Health Statistics Yearbook.

## 3. Results

**Figure 1:**
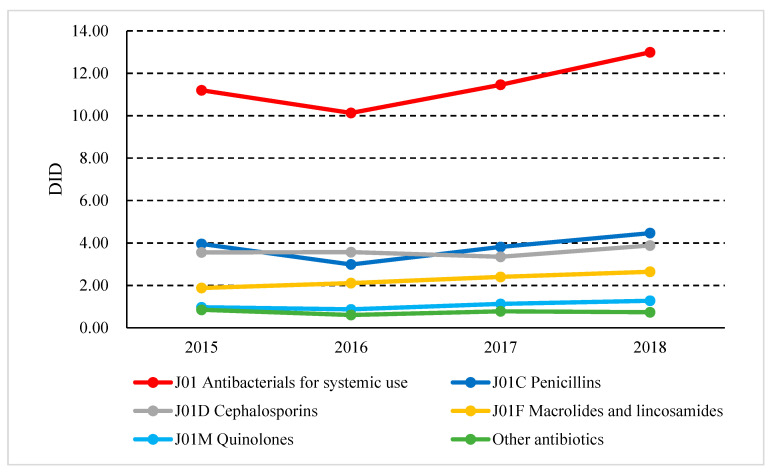
Antibiotic consumption in Shaanxi Province, western China, 2015–2018. (DDDs, defined daily doses; DID, DDDs per 1000 inhabitants per day).

In 2018, the consumption of antibiotics was 12.99 DID in Shaanxi Province. From 2015 to 2018, consumption decreased from 11.20 DID in 2015 to 10.13 DID in 2016, and then continued to rise, reaching 12.99 DID in 2018. The most used antibiotics in 2018 were penicillins with 4.47 DID, followed by cephalosporins (3.88 DID), macrolides (2.64 DID), and quinolones (1.27 DID) (Figure 1).

**Figure 2:**
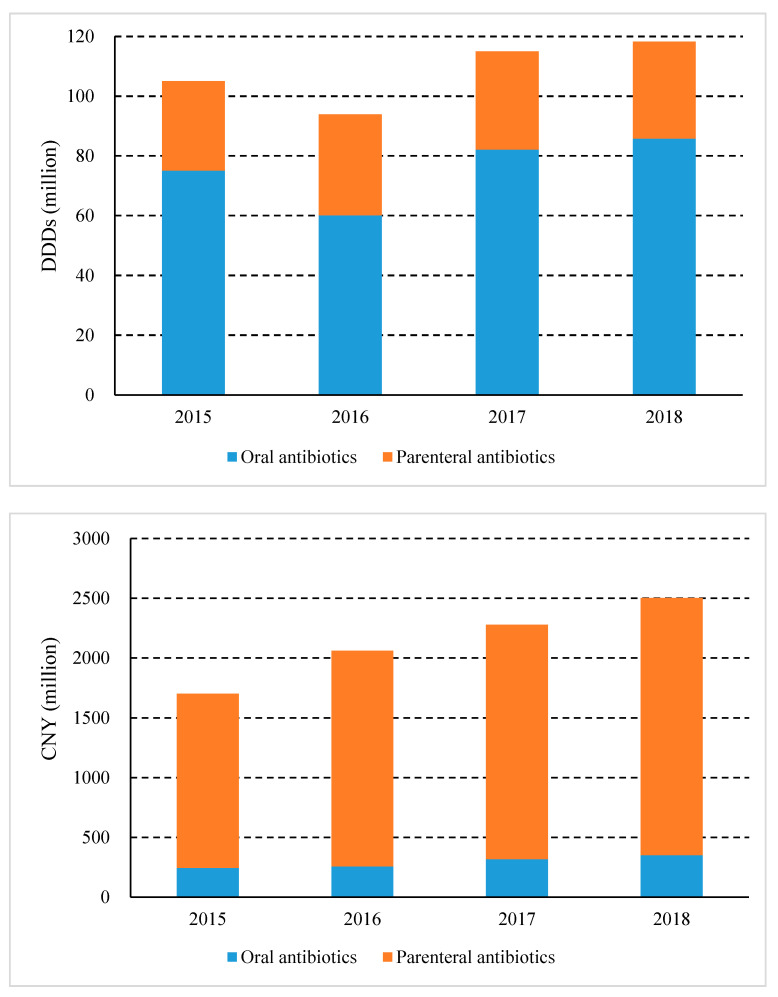
Consumption and expenditure of oral and parenteral antibiotics in Shaanxi Province, western china, 2015–2018. (DDDs, defined daily doses; CNY, Chinese Yuan).

**Figure 3:**
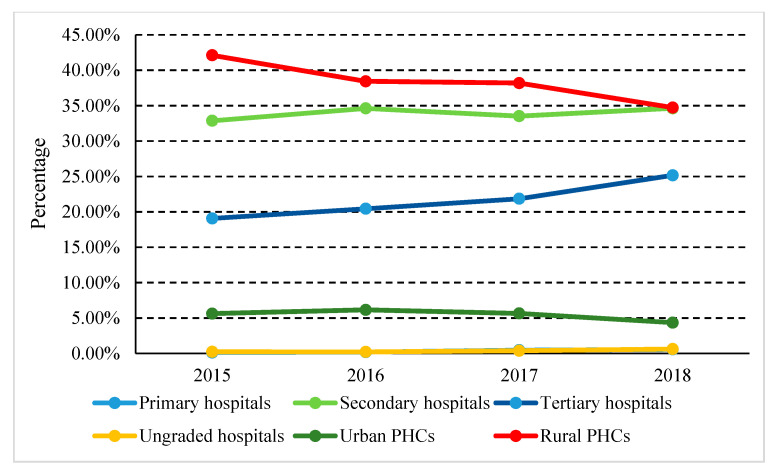
Proportional consumption (%) of parenteral antibiotics used in healthcare institutions in Shaanxi Province, western China, 2015–2018.

Parenteral antibiotic consumption accounted for 27.41% of the total consumption, while oral antibiotics accounted for 72.59% in 2018. However, parenteral and oral antibiotics, respectively, accounted for 85.98% and 14.02% of medicine expenditure. From 2015 to 2018, the consumption of parenteral antibiotics decreased from 28.57% to 27.41%, but expenditure remained almost unchanged (~86%) (Figure 2). Among them, the proportion of parenteral antibiotic consumption in rural PHCs decreased from 42.11% in 2015 to 34.72% in 2018 and urban PHCs dropped from 5.62% to 4.43% during the same period (Figure 3).

**Figure 4:**
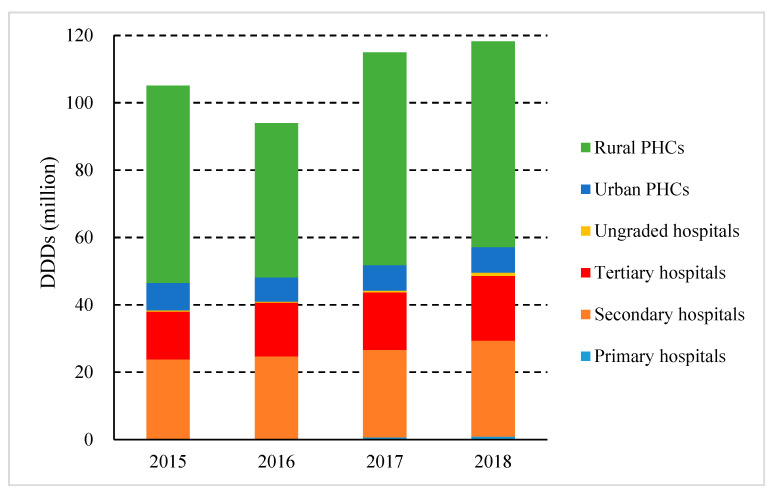

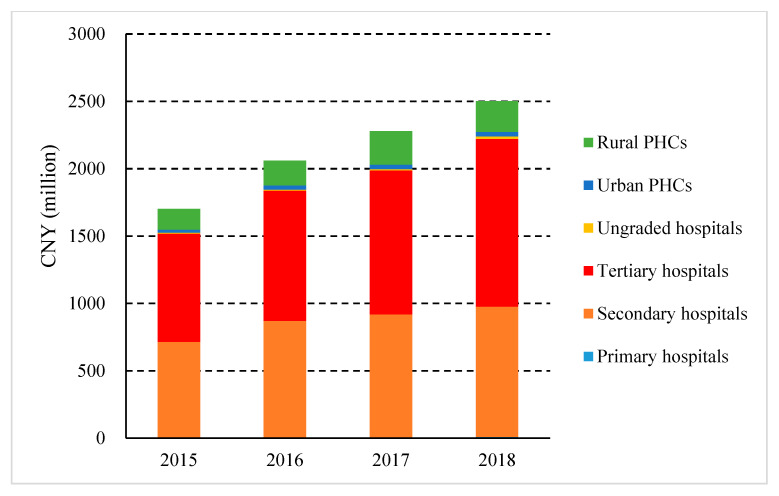
Consumption and expenditure of antibiotics in different healthcare institutions in Shaanxi Province, western China, 2015–2018. (DDDs, defined daily doses; CNY, Chinese Yuan).

In analyzing antibiotic use from the healthcare institutions studied (Figure 4), it was found that the largest consumption of antibiotics in Shaanxi Province was in rural PHCs (51.67%), followed by secondary hospitals (24.10%), tertiary hospitals (16.25%), and urban PHCs (6.38%). In terms of expenditure, secondary and tertiary hospitals spent 38.64% and 49.78% of the total expenditure, rural PHCs accounted for 9.12%, and urban PHCs accounted for 1.32%. During the study period, antibiotic consumption decreased from 55.76% to 51.67% in rural PHCs, while a decrease from 7.70% to 6.38% was observed in urban PHCs. Contrastingly, there was an increase in both secondary (22.53% to 24.10%), and tertiary hospitals (13.49% to 16.25%).

**Figure 5:**
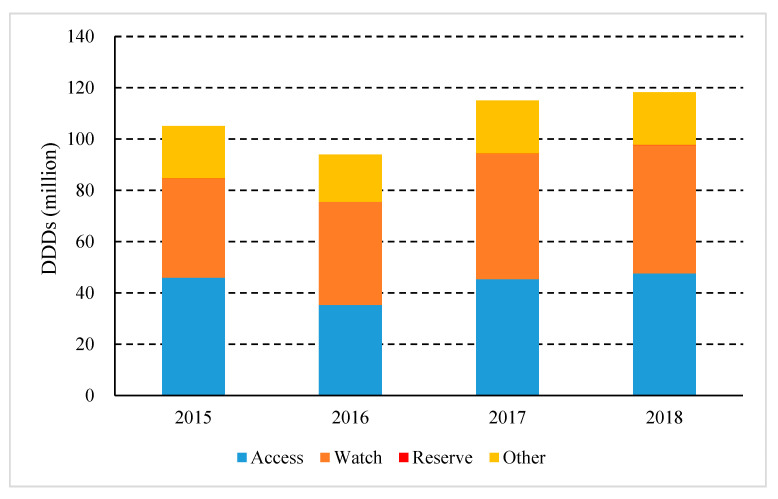

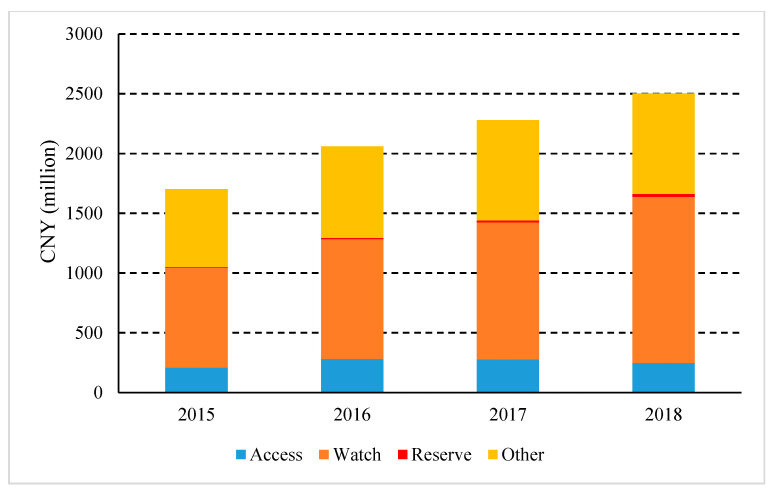
Consumption and expenditure of antibiotics by AWaRe categorization in Shaanxi Province, western China, 2015–2018. (DDDs, defined daily doses; CNY, Chinese Yuan; AWaRe, the “Access, Watch and Reserve” classification)

**Figure 6:**
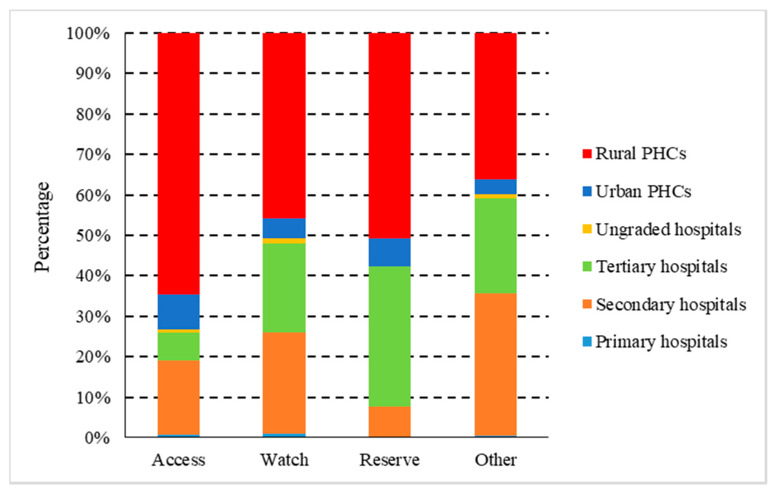
Proportional consumption (%) of antibiotics used in healthcare institutions by AWaRe categorization in Shaanxi Province, western China, 2015–2018. (AWaRe, the “Access, Watch and Reserve” classification.)

According to the AWaRe classification, the antibiotics of the Watch group were the most consumed (42.28%), followed by the Access group (40.31%) and the Reserve group (0.11%). Expenditure on the Watch group accounted for 55.57%, followed by the Access group (9.91%), and the Reserve group (1.01%). From 2015 to 2018, the consumption of the Access group decreased from 43.76% to 40.31%; the Watch and the Reserve group increased from 36.72% and 0.06% to 42.28% and 0.11% accordingly (Figure 5). In 2018, the Access, Watch, and Reserve groups of antibiotics were consumed the most in rural PHCs, accounting for 65.51%, 45.78%, and 50.68% of total consumption, respectively (Figure 6).

Comparing the 2015–2018 data of Shaanxi Province with the ESAC data (Table 2), J01C (penicillins) were used less not only in absolute terms (DID) but also in relative terms as a percentage of total antibiotic procurement. J01M (quinolones) were used in the middle or high range of the compared countries. J01D (cephalosporins) were used in large amounts, which were higher than at least three quartiles of the compared countries. In terms of relative usage, the proportion of J01MA (fluoroquinolones) use was higher than 50% of the compared countries. J01 (DD + DE) (third and fourth generation cephalosporins) were widely used and higher than most of the compared countries. The ratio of broad/narrow spectrum antibiotics increased yearly, but it was still within the range of the top 25–50% when compared with European countries. Seasonal variations in both total antibiotics and quinolones fluctuated in the middle range (20–50%).

## 4. Discussion

This study presents empirical evidence on trends in antibiotic consumption and expenditure from the healthcare institutions in Shaanxi Province’s centralized procurement platform database between 2015 and 2018. We found that the consumption of antibiotics in Shaanxi Province was 12.99 DID in 2018, which was in the top 22% compared with 65 countries published in the WHO Antimicrobial Consumption Monitoring Report [27], similar to Latvia (13.3 DID), lower than the consumption of Shanghai in 2014 (17.8 DID) and Shandong in 2016 (13.802 DID) [28,29], and higher than the consumption of primary medical and health service institutions in Hubei in 2017 (9.1 DID) [30]. The consumption of antibiotics showed an overall upward trend during the study period. Since 2014, the Chinese government has introduced antibiotics use policies that limit their sales from community pharmacies in Shaanxi Province [31]. This had led to a drastic decrease in the practice of dispensing antibiotics without a prescription from community pharmacies. Hence, most patients had to get their antibiotics from hospitals, which may explain the increase in antibiotics from these health facilities.

Our results showed a decrease in parenteral antibiotic consumption between 2015 and 2018, and the main contribution of the decline was PHCs. PHCs mainly provide outpatient services in China, so the control target of parenteral antibiotic proportion can refer to the European average (2%) for outpatient medication [32]. At present, the proportion of parenteral antibiotic use in Shaanxi Province is far from reaching this standard value. In China, injectable drugs are often considered by patients to be more potent and effective than oral drugs, which also puts pressure on doctors to prescribe [33]. This may be a factor that has contributed to the consistently high rate of antibiotic injection. However, a decline in urban and rural PHCs consumption of parenteral antibiotics was also observed, which may be due to the introduction of local restriction policies. In 2016, Shaanxi Province introduced a policy that primary healthcare institutions must conduct review and filing before using parenteral medications [34]. It is only upon approval that infusion medication can be administered. Additionally, the County Health Bureau uniformly carried out a certification training program for rural doctors on intravenous infusion and emergency treatment and periodically assesses their knowledge and ability in relation to infusion and adverse drug reactions. Only those with a pass in the examination are allowed to carry out infusion services. These measures have led to a significant reduction in the use of parenteral antibiotics in PHCs.

According to the WHO Antimicrobial Consumption Monitoring Report including data from 65 countries [27], the consumption of the Watch group of antibiotics in healthcare institutions in Shaanxi Province was higher, while that of the Access group and the Reserve group were lower. Compared with European regions, the consumption of the Access group and the Reserve group was lower than the median of the European region (40% vs. 56%, 0.1% vs. 0.2%), respectively, while the consumption of the Watch group was higher than the median of European region (42% vs. 29%). By quantifying the use of antibiotics in each AWaRe category to measure the utilization of antibiotics, it is possible to infer the overall quality and differences in antibiotic use between countries. The overuse of Watch antibiotics was clearly found, and the reduction in Watch antibiotics can be determined as the target of antibiotic stewardship intervention. Our study found that the consumption of the Watch group was mainly azithromycin, levofloxacin, ceftriaxone; and the consumption of Reserve group was only three types, which were fosfomycin, Linezolid and tigecycline; these drugs should be clinically evaluated and used with caution. The Watch group is mainly consumed in rural PHCs (65.51%), followed by secondary hospitals, urban PHCs, and tertiary hospitals, which are 18.30%, 8.84%, and 6.94%, respectively, and the Reserve group is mainly used in rural PHCs (50.68%), followed by tertiary hospitals (34.58%), secondary hospitals (7.41%), and urban PHCs (7.08%). It is worth noting that the consumption of the Watch and the Reserve group in rural PHCs is relatively high. Therefore, we should strengthen the management of antibiotics in PHCs, and reduce the use of unnecessary antibiotics.

There are problems with the quality of antimicrobial use in healthcare institutions in Shaanxi Province. Compared with 31 European countries [26], penicillin antibiotics were less used, while quinolones and cephalosporins were used more frequently, especially third and fourth generation cephalosporins. Chinese clinicians tend to use cephalosporins, which is due to a lack of professional competence for microbiological testing [35]. The use of third and fourth generation cephalosporins as broad-spectrum antibiotics should be reduced, but their usage proportion accounts for nearly half of the use of cephalosporins, which is also found in Hubei (primary healthcare institutions) and some countries in Eastern Europe and Central Asia [30,36]. Decreasing the use of third-generation cephalosporins can increase the drug susceptibility of seriously ill patients and reduce the mortality caused by drug resistance [37].

At present, the use of antibiotics in PHCs in Shaanxi Province has been effectively controlled, but the antibiotic consumption in secondary and tertiary hospitals continued to rise. Hence, there is a need for strengthening the management and use of antibiotics in secondary and tertiary hospitals. Furthermore, the monitoring system of key drugs, the warning system of the unusual application of drugs, and the review system of prescription should be optimized. It is advisable to focus on the monitoring of drugs with high prices and large dosages, and to inform and interview the relevant physicians with unreasonable antibiotic prescriptions. At the same time, doctors should also be regularly trained in the rational use of antibiotics to make them clear about the indications and precautions relating to antibiotics, thereby changing the behavior of some physicians who use antibiotics unreasonably. Part of the public in China has insufficient knowledge about drug use and lacks awareness of the rational use of antibiotics. Therefore, it is necessary to carry out publicity and education activities on rational antibiotic use. Health administration departments, healthcare institutions, scientific research institutes, and related associations at various levels can cooperate to carry out a wide variety of educational activities on rational antibiotic use, so as to provide a guarantee for the rational use of antibiotics by the public. Physicians and pharmacists need to work together to pay attention to the side effects of antibiotics and reduce the use of unnecessary antibiotics, especially the use of the third and fourth generation cephalosporins and the Watch group antibiotics, as well as the high proportion of injection use.

This study has several limitations which must be considered while interpreting the findings. First, the purchase data cannot reflect the length of time that the healthcare institution stores the drug as an inventory after purchasing the drug and the number of expired or discarded drugs during the storage of the drug. Second, the healthcare institution’s drug purchase records cannot distinguish between outpatient medication and inpatient medication, which limits the differences in antibiotic use between outpatient and inpatient scenarios. Third, as we do not have data on the use of antibiotics in community pharmacies, there will be an underestimation in calculating the population’s medication intensity based solely on the use of healthcare institutions. Despite these limitations, this study is the first report on antibiotic use in western China, and the procurement data of healthcare institutions can largely reflect the real situation of antibiotic use.

## 5. Conclusions

The total antibiotic consumption in Shaanxi Province had been on an upward trend, and the patterns of antibiotic use were not sufficiently justified to conclude that it was rational. This is partly because there was high preference for third and fourth generation cephalosporins and for antibiotics in the Watch group. It is necessary for the government to strengthen the management of the use of antibiotics, improve the professional level of physicians in healthcare institutions, and strengthen publicity and education to improve the rational use of antibiotics. More detailed prescription information and clinical data are needed to study and evaluate the use of antibiotics in the future.

## Figures and Tables

**Table 1:** Number of healthcare institutions in Shaanxi Province, western China, 2015–2018.

Year	Primary Hospitals	Secondary Hospitals	Tertiary Hospitals	Ungraded Hospitals	Urban PHCs	Rural PHCs
2015	9	217	48	15	149	1220
2016	9	224	47	14	150	1213
2017	62	235	48	28	162	1264
2018	54	231	48	23	155	1225

PHCs, primary healthcare centers.

**Table 2:** Quality indicators for antibiotic use in Shaanxi Province in comparison with 31 European countries, 2015–2018.

Year		2015	2016	2017	2018
Absolute Use	J01_DID	11.20	10.13	11.46	12.99
	J01C_DID	3.95	2.99	3.81	4.47
	J01D_DID	3.55	3.57	3.35	3.88
	J01F_DID	1.88	2.11	2.40	2.64
	J01M_DID	0.97	0.87	1.13	1.27
Relative Use	J01CE_%	1.19%	1.21%	1.18%	1.01%
	J01CR_%	4.82%	6.30%	7.59%	8.07%
	J01DD + DE_%	11.63%	13.41%	12.84%	13.42%
	J01MA_%	7.67%	7.52%	9.66%	9.72%
Broad/Narrow	J01_B/N	3.41	5.74	5.76	6.24
Seasonal Variations	J01_SV	34.14%	8.30%	21.04%	N/A
	J01M_SV	9.44%	1.36%	11.09%	N/A

The color indicates the quartile: 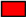 values within the fourth quartile (i.e., p75–p100), 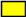 values within the third quartile (i.e., p50–p75), 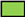 values within the second quartile (i.e., p25–p50), 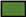 values within the first quartile (i.e., p0–p25).
